# Application of Fast Dynamic Allan Variance for the Characterization of FOGs-Based Measurement While Drilling

**DOI:** 10.3390/s16122078

**Published:** 2016-12-07

**Authors:** Lu Wang, Chunxi Zhang, Shuang Gao, Tao Wang, Tie Lin, Xianmu Li

**Affiliations:** 1 Key Laboratory of Inertial Technology, Institute of Opto-electronics Technology, School of Instrument Science and Opto-electronics Engineering, Beihang University, Beijing 100191, China; wanglu@buaa.edu.cn (L.W.) zhangchunxi@buaa.edu.cn (C.Z.); opticlin@163.com (T.L.); lixianmu@buaa.edu.cn (X.L.); 2 Beijing Aerospace Times Laser Inertial Technology Company, Ltd., Beijing 100094, China; wtwdqa@163.com

**Keywords:** dynamic stability, FOGs-MWD, dynamic Allan variance, fast algorithm, discontinuous data

## Abstract

The stability of a fiber optic gyroscope (FOG) in measurement while drilling (MWD) could vary with time because of changing temperature, high vibration, and sudden power failure. The dynamic Allan variance (DAVAR) is a sliding version of the Allan variance. It is a practical tool that could represent the non-stationary behavior of the gyroscope signal. Since the normal DAVAR takes too long to deal with long time series, a fast DAVAR algorithm has been developed to accelerate the computation speed. However, both the normal DAVAR algorithm and the fast algorithm become invalid for discontinuous time series. What is worse, the FOG-based MWD underground often keeps working for several days; the gyro data collected aboveground is not only very time-consuming, but also sometimes discontinuous in the timeline. In this article, on the basis of the fast algorithm for DAVAR, we make a further advance in the fast algorithm (improved fast DAVAR) to extend the fast DAVAR to discontinuous time series. The improved fast DAVAR and the normal DAVAR are used to responsively characterize two sets of simulation data. The simulation results show that when the length of the time series is short, the improved fast DAVAR saves 78.93% of calculation time. When the length of the time series is long ($6\times 10^{5}$ samples), the improved fast DAVAR reduces calculation time by 97.09%. Another set of simulation data with missing data is characterized by the improved fast DAVAR. Its simulation results prove that the improved fast DAVAR could successfully deal with discontinuous data. In the end, a vibration experiment with FOGs-based MWD has been implemented to validate the good performance of the improved fast DAVAR. The results of the experience testify that the improved fast DAVAR not only shortens computation time, but could also analyze discontinuous time series.

## 1. Introduction

Gyroscopes are sensors that are appropriate for a wide variety of applications in the inertial navigation scope. The fiber optic gyroscope (FOG), which has become mainstream in inertial navigation systems, is utilized to determine altitude for satellites and missiles [1]. The Ring Laser gyroscope is the ideal angular sensor for high-precision and long-endurance inertial navigation systems [2]. The MEMS (Micro-electromechanical systems) gyroscope is widely used in cheap and small applications, such as unmanned aerial vehicles [3]. The measurement stability of gyroscopes can vary over time due to several factors, such as temperature, humidity, radiation, and ageing [4]. A slight instability in the gyroscopes will lead to an increase in measurement error. FOGs in measurement while drilling (MWD) systems work even in poor conditions. Their temperature increases as the drilling depth increases [5]. Additionally, FOGs withstand shock when drilling the oil hole. Therefore, it is fundamental to evaluate how the measurement stability of gyroscopes behaves over time. Allan variance is a common and standard method to analyze gyroscopes [6], but it cannot describe the dynamic characteristic. In 2003, in order to track and reveal the anomaly in the atom clock behavior, Galleani and Tavella developed Dynamic Allan Variance (DAVAR) [7]. Although it is an extension of Allan variance, DAVAR is a new method that can track and describe the non-stationary characteristics of time series [8,9,10,11,12,13]. Li, Zhang, and Wei extended the DAVAR to diagnose the non-stationary of gyroscope [14,15,16,17,18]. Since the DAVAR requires instant calculation of Allan variance, its calculation burden is a critical shortcoming [19,20,21,22]. Wang and Zhang developed a fast algorithm for DAVAR to simply the DAVAR algorithm and accelerate the calculation speed [23].

An MWD system keeps working for several days underground. The output signal of the gyroscopes in the MWD system would be interrupted due to error in the mud pulse communication or sudden power outages. In this case, both the classical DAVAR and the fast DAVAR algorithms are unable to deal with discontinuous time series. Hence, in order to deal with the discontinuous gyroscope data, a further improvement is made on the basis of the fast DAVAR. The recursion characteristic of the fast DAVAR will judge whether the data contains unreadable code or not. It makes the fast DAVAR available to deal with discontinuous gyroscope data. Thus, the improved fast algorithm of the DAVAR is more valuable in engineering applications.

This article is organized as follows. In Section 2, the structure of the FOG-based MWD and its working characteristics are introduced. In Section 3, the theory of Allan variance is briefly presented. In Section 4, we sum up the calculation process of the classical DAVAR. In addition, a 2D diagram illustrating the noise characteristics of FOGs is reported. In Section 5, the fast algorithm of the DAVAR is derived step by step. In Section 6, a further improvement has been made and the improved fast DAVAR is extended to discontinuous data. In Section 7, in order to test that the improved fast DAVAR is superior to the classical method, three sets of simulation data have been analyzed by the two methods. In Section 8, a set of discontinuous time series collected from a vibration experiment with the FOGs-based MWD is analyzed by the fast DAVAR. The conclusions are presented in Section 9.

## 2. Structure of the FOG-Based MWD

**Figure 1:**
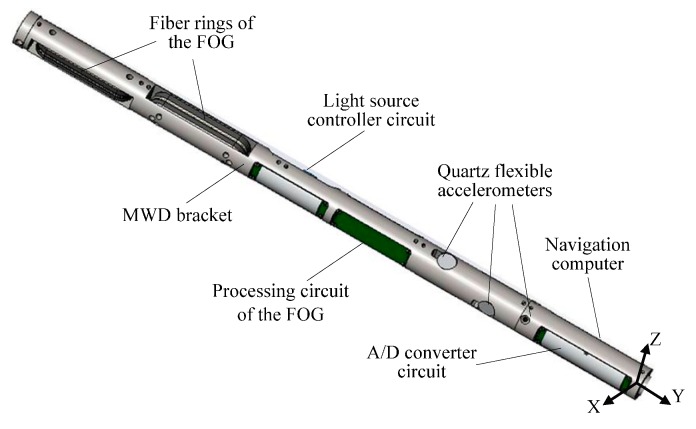
3D graphic model of the MWD.

**Figure 2:**
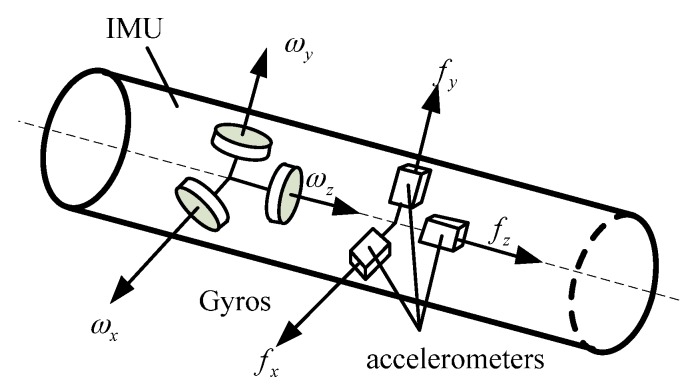
Sensors of IMU.

The MWD prototype utilizes a FOG-based IMU, as shown in Figure 1. MWD is composed of three-axis FOGs sharing one laser light source, three quartz flexible accelerometers with one A/D converter circuit, a navigation computer, and a mechanical bracket to support the above units [24]. All the components are orthogonally mounted along the lengthwise axis of the MWD so that the diameter can be minimized to satisfy the demands of a borehole environment. The FOGs and accelerometers are arranged in three mutually orthogonal directions, as shown in Figure 2. The three-axis FOGs measure the angular velocity of the carrier, while three-axis accelerometers provide the 3D acceleration measurements of the body. The navigation computer is mainly utilized to collect all sensor data, pre-process the data, and run the navigation algorithm.

Due to the particularity of the working environment in oil wells, the FOGs-based MWD working manner and characteristics are different from other application fields such as aerospace, aviation, and land navigation. Comprehensive analysis shows that the detection and location of oil wells have the following characteristics:

(1) Long working hours: the FOGs-based MWD system keeps working underground for more than 200 h. Putting the MWD system down into the well and pulling it up to the ground costs too much time and money [25].
(2) Strong vibration: FOGs in MWD system hold up to strong shocks due to the obstruction of the underground stone.
(3) High rotation speed. FOGs in MWD systems work with a high-speed rotation up to 300 rounds per second.
(4) High Temperature. The temperature of the working environment increases with the increase in the drilling depth at a rate of 30 °C/km.
(5) Data loss. The data transmission method of the MWD system is mud pulse. When drilling the oil well, the mud pulse transmits the data collected downhole to the monitoring equipment on the ground. Its transfer rate is low. Its bit error rate is high and the reliability is very low.
(6) Eclectic battery-powered. A battery provides power to support the MWD system working underground.

When drilling the oil well, FOGs’ signal in MWD system is non-stationary, since the FOGs can be easily influenced by the increasing temperature, humidity, radiation, and sudden power failure. Occasionally, the data received aboveground appears to be discontinuous. Even a slight variation in the gyroscope stability can generate significant measurement error. Accordingly, it is crucial to identify the dynamic instability of the FOGs in MWD.

## 3. Allan Variance

FOGs sense the angle velocity. Their output signal is the angle velocity ω(t). Allan variance [26] has commonly been used to evaluate the stability of FOG signal ω(t). The standard concept of the Allan variance is:

(1) $$\sigma_{\omega }^{2}(\tau )=\frac{1}{2}\langle {\left(\bar{\omega }(t+\tau )-\bar{\omega }(t)\right)}^{2}\rangle ,$$

where τ is the observation interval and 〈 〉 indicates a time averaging. The average of ϖ(t) is given by

(2) $$\bar{\omega }(t)=\frac{1}{\tau }\int_{t}^{t+\tau }\omega (u)du,$$

where u is the integral variable. Equation (1) is the definition of Allan variance for continuous data. If the Allan variance is to be estimated on discrete samples ω[n] whose total number is *N*, *t* is sampled as

(3) $$t=n\tau_{0},(n\;=\;1,2,\cdots N),$$

where $\tau_{0}$ is the sampling interval. Consequently, the observation interval τ is discretized is

(4) $$\tau =k\tau_{0},(k=1,2,\cdots \frac{N}{2}).$$

For the discrete data, the Allan variance becomes

(5) $$\sigma_{\omega }^{2}(k)=\frac{1}{2k^{2}\tau_{0}^{2}}\frac{1}{N-2k}\sum_{n=0}^{N-2k-1}{\left(\bar{\omega }[n+k]-\bar{\omega }[n]\right)}^{2}).$$

The average of ϖ[n] is given by

(6) $$\bar{\omega }[n]=\frac{1}{k}\sum_{i=n}^{n+k-1}\omega [i].$$

Therefore, according to the different observation interval k, the corresponding Allan variance can be obtained.

Ng [26] shows that a unique relationship existing between $\sigma_{\omega }^{2}(\tau )$ and the power spectral density (PSD) of the intrinsic random processes. This relationship is

(7) $$\sigma_{\omega }^{2}(\tau )=4\int_{0}^{\infty }S_{\Omega }(f)\frac{\sin^{4}(\pi f\tau )}{{\left(\pi f\tau \right)}^{2}}du,$$

where $S_{\Omega }(f)$ is the PSD of the random process Ω(t), namely the instantaneous output rate of the gyro.

Equation (7) is the focal point of the Allan variance technique. The PSD of any physically meaningful random process can be substituted in the integral and an expression for the Allan variance $\sigma_{\omega }^{2}(\tau )$ as a function of cluster length τ is identified [26,27]. Equation (7) states that the Allan variance is proportional to the total power output of the random process when passed through a filter with the transfer function $\frac{\sin^{4}(\pi f\tau )}{{\left(\pi f\tau \right)}^{2}}$. This particular transfer function is the result of the method used to create and operate on the cluster. It is seen from Equation (7) that the filter bandpass depends on τ. This suggests that different types of random processes can be examined by adjusting the filter bandwidth, namely by varying τ [28].

**Figure 3:**
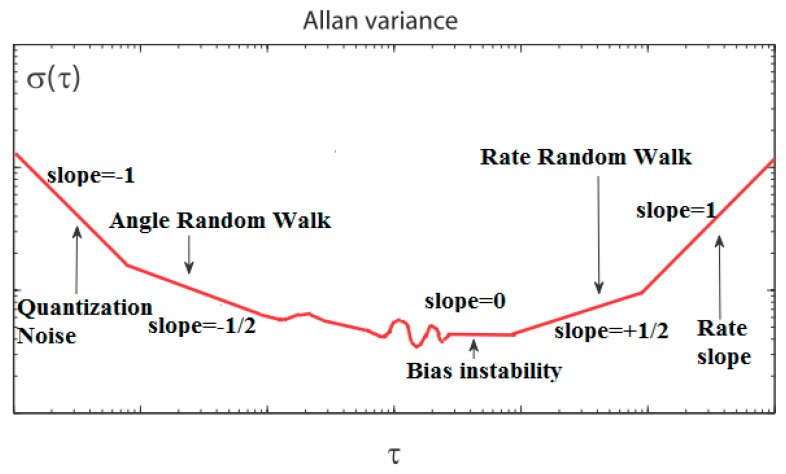
Sample plot of Allan variance analysis results.

Consequently, since $\sigma_{\omega }^{2}(\tau )$ is a measurable quantity, a plot of $\sigma_{\omega }^{2}(\tau )$ versus τ provides a direct indication of the types of random processes that exist in the gyro data. A log-log plot of the square root of the Allan variance $\sigma_{\omega }(\tau )-\tau$ provides a means of identifying and quantifying various noise terms that exist in the gyro sensor data, as Figure 3 shows. Table 1 gives a representation of noise terms using Allan variance.

In general, any number of the random processes discussed above can be present in the data. Thus, a typical Allan variance plot looks like the one shown in Figure 3. Different noise terms appear in different regions of τ. This allows for easy identification of the various random processes that exist in the gyro data. If it can be assumed that the existing random processes are all statistically independent, then it can be shown that the Allan variance at any given τ is the sum of the Allan variances due to the individual random processes at the same τ [27]. In other words,

(8) $$\begin{array}{l} \sigma^{2}(\tau )=\sigma_{Q}^{2}(\tau )+\sigma_{N}^{2}(\tau )+\sigma_{B}^{2}(\tau )+\sigma_{K}^{2}(\tau )+\sigma_{R}^{2}(\tau )\\ \;\;=\frac{3Q^{2}}{\tau^{2}}+\frac{N^{2}}{\tau }+\frac{2B^{2}}{\pi }\ln2+\frac{K^{2}\tau }{3}+\frac{R^{2}\tau^{2}}{2} \end{array},$$

where $\sigma_{Q}^{2}(\tau ),\sigma_{N}^{2}(\tau ),\sigma_{B}^{2}(\tau ),\sigma_{K}^{2}(\tau ),\sigma_{R}^{2}(\tau )$ represent Allan variance due to the individual random processes. Then the noise coefficient of the five noise terms can be obtained by a data fitting algorithm. When the dimension of FOG output data is in degrees per hour (°/h), the different noise terms are as follows:

(9) $$\begin{array}{l} N=\frac{\sqrt{C_{-1}}}{60}({}^{\circ}/h^{\frac{1}{2}})\\ K=60\sqrt{3C_{1}}({}^{\circ}/h^{\frac{3}{2}})\\ B=\frac{\sqrt{C_{0}}}{0.664}({}^{\circ}/h)\\ Q=\frac{10^{6}\pi \sqrt{C_{-2}}}{180\times 3600\times \sqrt{3}}(\prime \prime )\\ R=3600\sqrt{2C_{2}}({}^{\circ}/h^{2}) \end{array}.$$

## 4. Dynamic Allan Variance

### 4.1. Computation Process of Dynamic Allan Variance

The dynamic Allan variance (DAVAR) is an extension of the Allan variance. Firstly, truncate the data with a rectangular window centered at a given time point. Secondly, calculate the Allan variance of the truncated data. Then repeat the above two steps at every time epoch. In the end, by collecting all the variances obtained at every epoch and plotting the results in a signal 3D graph [8], we can obtain the DAVAR. The detailed computation process can be found in [9]. For continuous-time signals, the DAVAR could be obtained using Equation (10):

(10) $$\sigma_{\omega }^{2}(t,\tau )=\frac{1}{2\tau^{2}(N_{w}-2\tau )}\int_{t-\frac{N_{w}}{2}+\tau }^{t+\frac{N_{w}}{2}-\tau }\left(\varpi (u+\tau )-\varpi (u\right){)}^{2}du,$$

where $N_{w}$ is the length of the truncation window, *t* is the analysis time point, τ is the observation interval, and $\sigma_{\omega }^{2}(t,\tau )$ is the DAVAR.

The DAVAR given in Equation (10) is continuous both in *t* and τ. For discrete time signals, by sampling the integral variable *u* and the observation interval τ as follows,

(11) $$u=m\tau_{0},(m=\frac{N_{W}}{2},2,\cdots N-\frac{N_{W}}{2}),\;\tau =k\tau_{0},(k=1,2,\cdots \frac{N_{W}}{2}),$$

we can obtain the standard notation of discrete-time signals.

(12) $$\sigma_{\omega }^{2}[n,k]=\frac{1}{2k^{2}\tau_{0}^{2}}\frac{1}{N_{w}-2k}\times \sum_{m=n-N_{w}/2}^{n+N_{w}/2-2k-1}{\left(\varpi [m+k]-\varpi [k]\right)}^{2},$$

where $\tau_{0}$ is the sampling interval, $k\tau_{0}$ is the observation interval of the discrete time, and *N* is the available number of ω(n). $N_{w}$ is the length of the analysis window. $N_{w}$ is assumed to be even.

### 4.2. 2D Description of FOG Noise Terms

The DAVAR is simply obtained by sliding the Allan variances on the time series. At each given time *t*, Equation (8) still holds true. Therefore, the Allan variance at any given time can be written as Equation (13):

(13) $$\sigma^{2}(t,\tau )=\sigma_{Q}^{2}(t,\tau )+\sigma_{N}^{2}(t,\tau )+\sigma_{B}^{2}(t,\tau )+\sigma_{K}^{2}(t,\tau )+\sigma_{R}^{2}(t,\tau ).$$

**Figure 4:**
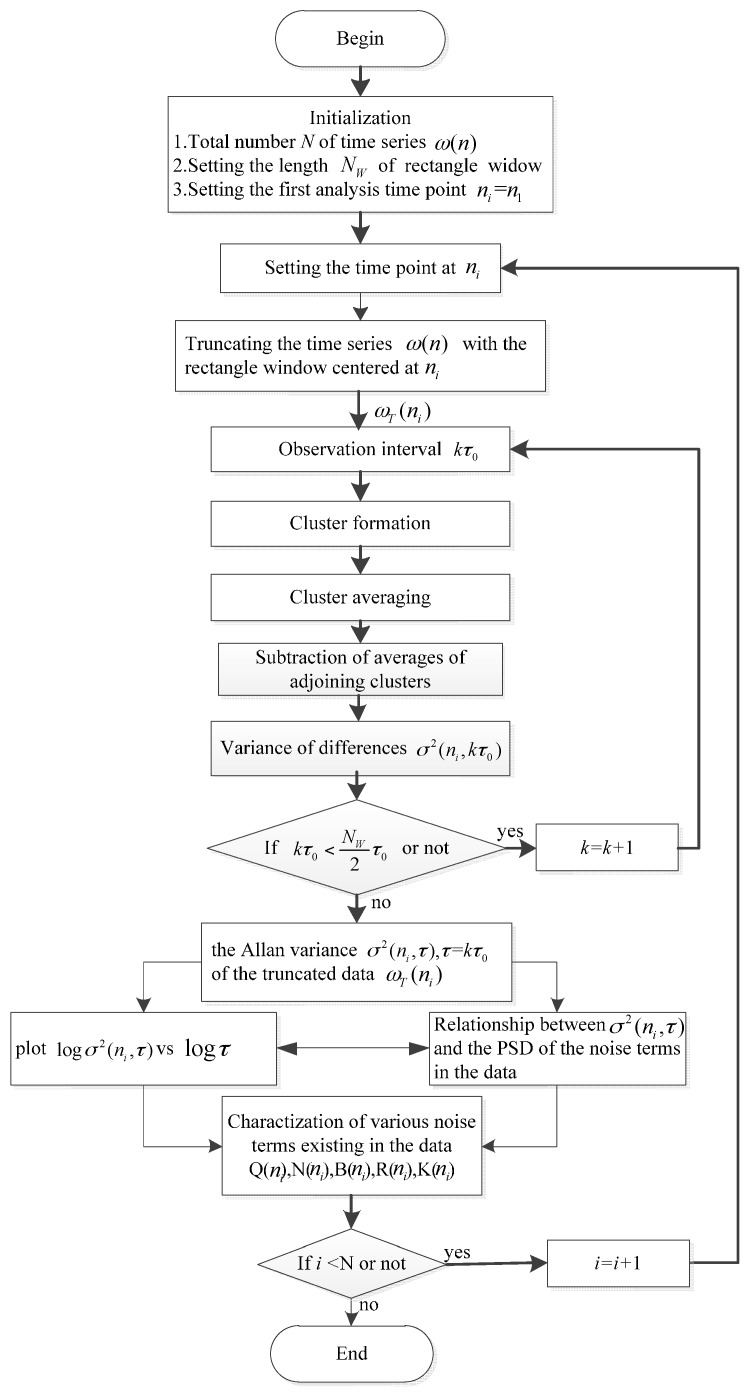
Flow chart of dynamic Allan variance.

At a given time *t*, there is a σ(t,τ)-τ curve. Correspondingly, we can obtain the coefficients *Q*(*t*), *N*(*t*), *B*(*t*), *K*(*t*), and *R*(*t*) of the five noise terms. Therefore, coefficients of the noise terms for all *t* could be obtained. In the end, by plotting *Q*(*t*), *N*(*t*), *B*(*t*), *K*(*t*), and *R*(*t*) according to the chronological order, the time-varying coefficients of FOGs noise terms can be characterized in a 2D diagram. Figure 4 gives a flowchart of the computation process of the DAVAR.

## 5. Fast Dynamic Allan Variance

Figure 4 shows that the DAVAR is obtained by computing the Allan variance at each analysis time epoch *t*. With the length of ω(t) increasing, it can result in a large computational burden. Therefore it is necessary to develop a fast algorithm for DAVAR. On the basis of the recursive characteristic of Allan variance, the recursive property of the DAVAR was found [29]. With this special characteristic, we can develop a recursive algorithm for the normal DAVAR, i.e., the fast DAVAR.

First define another time-series θ(t) connected to ω(t). The relationship between them is

(14) $$\omega (t)=\frac{d\theta (t)}{dt}$$

θ(n) denotes the angle, for which the dimension is given in degrees. Both ω(t) and θ(n) are random quantities. Then, substituting Equations (2) and (14) into Equation (10), Equation (10), which is the normal DAVAR, can be rewritten as

(15) $$\sigma_{\omega }^{2}(t,\tau )=\frac{1}{2\tau^{2}(N_{w}-2\tau )}\int_{t-\frac{N_{w}}{2}+\tau }^{t+\frac{N_{w}}{2}-\tau }{\left(\theta (u+2\tau )-2\theta (u+\tau )+\theta (u)\right)}^{2}du.$$

Using Equation (11), we can obtain the DAVAR for the discrete time gyro signal θ(n) as in Equation (16):

(16) $$\sigma_{\omega }^{2}[n,k]=\frac{1}{2k^{2}\tau_{0}^{2}}\frac{1}{N_{w}-2k}\sum_{m=n-N_{w}/2}^{n+N_{w}/2-2k-1}{\left(\theta [m+2k]-2\theta [m+k]+\theta [m]\right)}^{2}.$$

In order to describe the derivation simply, we name $\Delta_{k}[m]\;=\;\theta [m+2k]-2\theta [m+k]+\theta [m]$ as the discrete second order difference. Therefore Equation (16) can be rewritten as Equation (17):

(17) $$\sigma_{\omega }^{2}[n,k]=\frac{1}{2k^{2}\tau_{0}^{2}}\frac{1}{N_{w}-2k}\sum_{m=n-N_{w}/2}^{n+N_{w}/2-2k-1}\Delta_{k}^{2}[m].$$

Using Equation (17), we can obtain the DAVAR at time point *n* + 1 as follows:

(18) $$\sigma_{\omega }^{2}[n+1,k]=\frac{1}{2k^{2}\tau_{0}^{2}}\frac{1}{N_{w}-2k}\sum_{m=n+1-N_{w}/2}^{n+N_{w}/2-2k}\Delta_{k}^{2}[m].$$

$\sigma_{\omega }^{2}[n+1,k]$ can also be written with $\sigma_{\omega }^{2}[n,k]$, as Equation (19) shows.

(19) $$\begin{array}{l} \sigma_{\omega }^{2}[n+1,k]=\frac{1}{2k^{2}\tau_{0}^{2}}\frac{1}{N_{w}-2k}\sum_{m=n-N_{w}/2}^{n+N_{w}/2-2k-1}\Delta_{k}^{2}[m]+\frac{1}{2k^{2}\tau_{0}^{2}}\frac{1}{N_{w}-2k}(\Delta_{k}^{2}[n+\frac{N_{w}}{2}-2k]-\Delta_{k}^{2}[n-\frac{N_{w}}{2}])\\ \;=\sigma_{\omega }^{2}[n,k]+\frac{1}{2k^{2}\tau_{0}^{2}}\frac{1}{N_{w}-2k}(\Delta_{k}^{2}[n+\frac{N_{w}}{2}-2k]-\Delta_{k}^{2}[n-\frac{N_{w}}{2}]) \end{array}$$

At time *n*, the limit of *m* in discrete second-order difference $\Delta_{k}[m]$ lies in $\left[n-\frac{N_{w}}{2},n+\frac{N_{w}}{2}-2k-1\right]$. At time *n* + 1, the limit of *m* lies in $\left[n+1-\frac{N_{w}}{2},n+\frac{N_{w}}{2}-2k\right]$. Therefore, when we compute the $\sigma_{\omega }^{2}[n+1,k]$ based on the $\sigma_{\omega }^{2}[n,k]$, we just need to add the discrete second-order difference $\Delta_{k}^{2}[n+N_{w}/2-2k]$ and subtract the discrete second-order difference $\Delta_{k}^{2}[n-N_{w}/2]$. Equation (19) shows that the DAVAR at time *n* + 1 can be written as a function of the DAVAR at time *n*.

Since Equation (19) is a recursive algorithm for DAVAR, the initial value is required before the computation. On the assumption that the analysis starts at time $n_{1}$, the starting value is as in Equation (20):

(20) $$\sigma_{\omega }^{2}[n_{1},k]=\frac{1}{2k^{2}\tau_{0}^{2}}\frac{1}{N_{w}-2k}\sum_{m=n_{1}-N_{w}/2}^{n_{1}+N_{w}/2-2k-1}\Delta_{k}^{2}[m].$$

**Figure 5:**
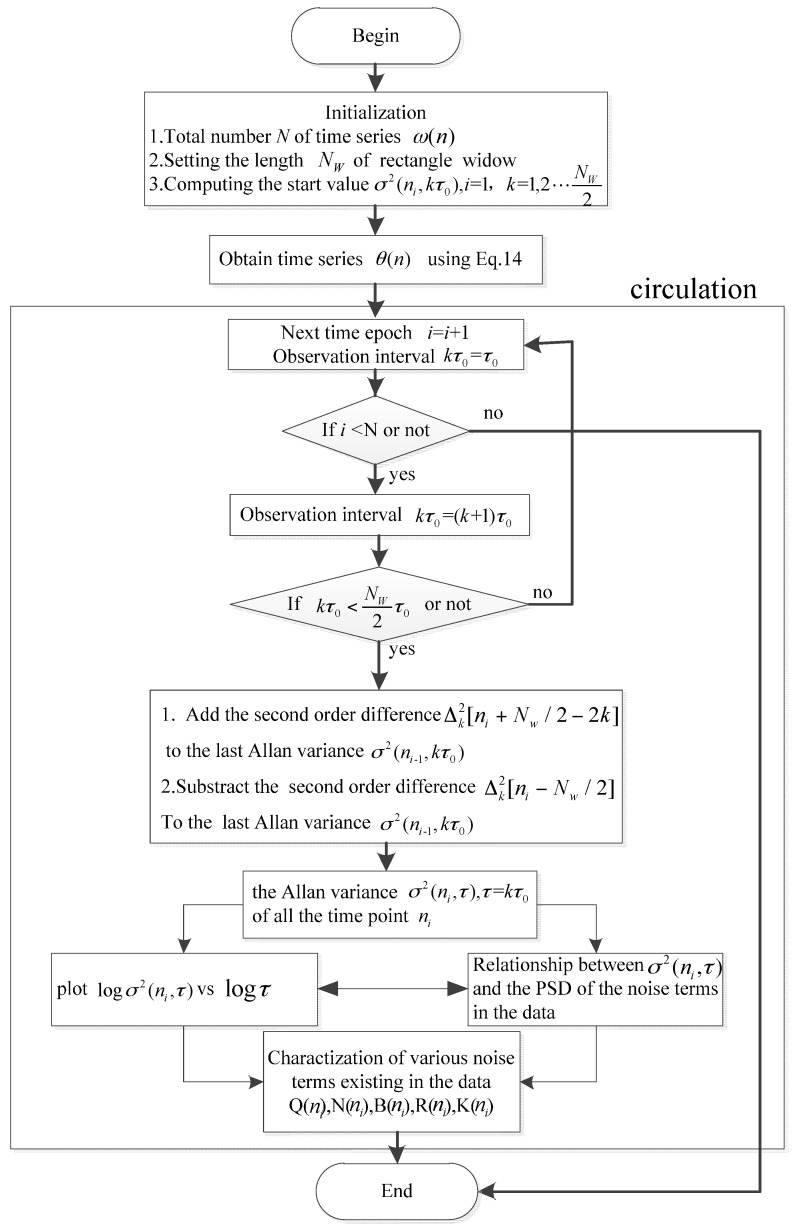
Flowchart of fast dynamic Allan variance.

In conclusion, with the recursive formula, it is not necessary to compute the Allan variance at every time epoch. The new DAVAR can be obtained based on the previous DAVAR. This can reduce the computation time dramatically. We named the recursive algorithm fast DAVAR and the ordinary one classical DAVAR. Figure 5 gives a flowchart of fast DAVAR.

## 6. Extension to Discontinuous Time Series

At present, when drilling oil wells, the MWD system utilizes the mud pulse to send the data from underground to the monitoring equipment on the ground. Its transfer rate and reliability are low while its bit error rate is high. Consequently, the data received on the ground often appears to be abnormal, such as with unreadable words or showing a loss of data in a certain period of time. So the data are discontinuous in the timeline. However, when meeting the discontinuous gyroscope data, both the classical DAVAR and the fast DAVAR are invalid. It is critical to further improve the algorithm of the DAVAR to deal with discontinuous time series. Based on the recursive character of the fast DAVAR algorithm, we make a further advance to extend the fast DAVAR to discontinuous time series.

Before computing $\sigma_{\omega }^{2}[n+1,k]$, we need to compute the discrete second-order difference $\Delta_{k}^{2}[n+N_{w}/2-2k]$ and $\Delta_{k}^{2}[n-N_{w}/2]$. For the given *n* and *k*, $\Delta_{k}^{2}[n+N_{w}/2-2k]$ and $\Delta_{k}^{2}[n-N_{w}/2]$ can be obtained by Equations (21) and (22):

(21) $$\Delta_{k}^{2}[n+\frac{N_{w}}{2}-2k]={\left(\theta [n+\frac{N_{w}}{2}]-2\theta [n+\frac{N_{w}}{2}-k]+\theta [n+\frac{N_{w}}{2}-2k]\right)}^{2}$$

(22) $$\Delta_{k}^{2}[n-\frac{N_{w}}{2}]={\left(\theta [n-\frac{N_{w}}{2}+2k]-2\theta [n-\frac{N_{w}}{2}+k]+\theta [n-\frac{N_{w}}{2}]\right)}^{2}.$$

At this time, we can judge whether $\theta [n+\frac{N_{w}}{2}]$, $\theta [n+\frac{N_{w}}{2}-k]$ and $\theta [n+\frac{N_{w}}{2}-2k]$ are unreadable words or not. If one of them is an unreadable word, we set $\Delta_{k}^{2}[n+N_{w}/2-2k]$ to zero and then continue the computation. $\Delta_{k}^{2}[n-N_{w}/2]$ could be obtained in the same way.

The recursion characteristic of the fast DAVAR will judge whether the data contains unreadable code or not. So the fast DAVAR could be extended to deal with discontinuous time series. We call this modified algorithm improved fast DAVAR.

## 7. Models and Simulations

In order to testify the proposal algorithm, two sets of simulation data are created. The model of the simulation data is shown as follows:

(23) x[n]=σ[n]f[n],

where f[n] is the white Gaussian noise [5], σ[n] is the standard deviation of f[n], and x[n] is simulating data of gyroscopes whose unit is °/h. The sampling interval of the two sets of simulation data is 0.01 s. The first simulation dataset $x_{1}[n]$ is stationary white Gaussian noise, whose standard deviation σ[n] is always equal to 1. The number of $x_{1}[n]$ is $L_{1}=6\times 10^{3}$ samples. So the time length of $x_{1}[n]$ is 60 s. The second one $x_{2}[n]$ is also white Gaussian noise, whose variance σ[n] increases with time linearly as Equation (24) shows. The number of $x_{2}[n]$ is $L_{2}=6\times 10^{5}$ samples. Thus the time length of $x_{2}[n]$ is 6000 s.

(24) $$\sigma [n]=1+\frac{10}{L_{2}}\times n$$

**Figure 6:**
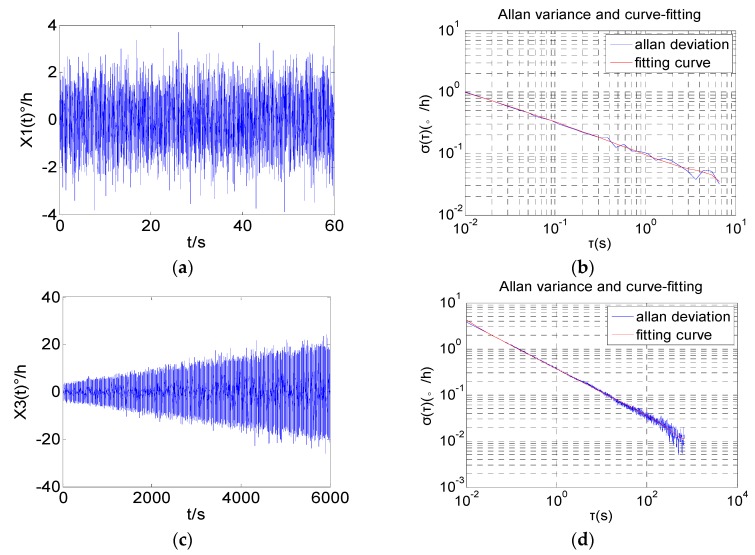
Two sets of simulation data. (**a**) white Gaussian noise $x_{1}[n]$; (**b**) Allan variance of $x_{1}[n]$; (**c**) white Gaussian noise with “increase” $x_{2}[n]$; (**d**) Allan variance of $x_{2}[n]$.

Two sets of simulation data are represented in Figure 6a,c. $x_{1}[n]$ is stationary. The amplitude of $x_{2}[n]$ increases with time.

Figure 6b,d exhibits the Allan variance of the two sets of simulated data. Because $x_{1}[n]$ is stationary white Gaussian noise, its slope is approximately −1/2 for all τ [30]. $x_{2}[n]$ is non-stationary white noise, but the shapes and slopes of Allan variance are the same as for the stationary white noise $x_{1}[n]$. Obviously, the Allan variance cannot track and reveal the non-stationary characteristics.

**Figure 7:**
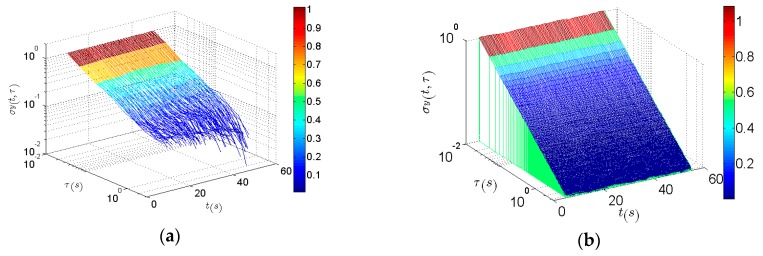

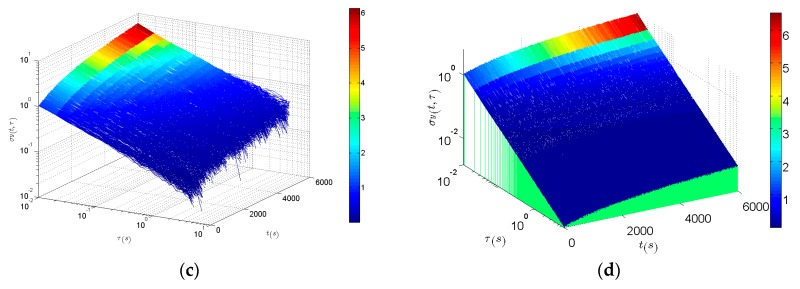
The classical DAVAR and the improved fast DAVAR. (**a**) DAVAR of white Gaussian noise $x_{1}[n]$; (**b**) The improved fast DAVAR of $x_{1}[n]$; (**c**) DAVAR of white noise with “increase” $x_{2}[n]$; (**d**) The improved fast DAVAR of $x_{2}[n]$.

Then the classical DAVAR and the improved fast DAVAR are applied to analyze the simulation data. The analysis results of the classical DAVAR Figure 6a,c and the improved fast DAVAR Figure 6b,d are shown in Figure 7.

With a rectangular window of *L* = 1000 samples and a step whose width is 30 samples, the analysis results of $x_{1}[n]$ are obtained. As can be seen in Figure 7a, both DAVAR methods are constant over time and have a typical white noise slope. The value of the improved fast DAVAR is similar to that of the classical DAVAR at fixed *t* and τ. The analysis results of $x_{2}[n]$ are obtained by a rectangular window of number $N_{W}$ = 2000 samples and a step whose width is 300 samples. In Figure 7b, the improved fast DAVAR and classical DAVAR reveal that noise increases linearly with time.

**Figure 8:**
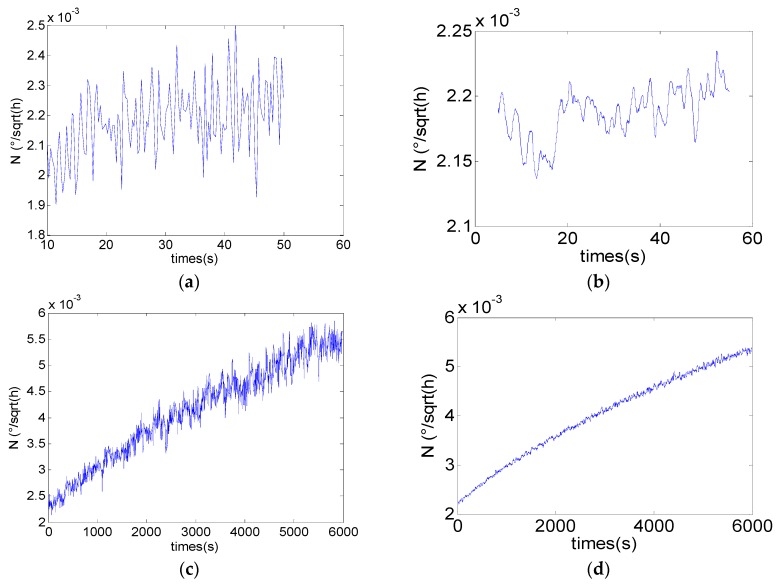
Angle random walk of the simulation data. (**a**,**b**) Angle random walk of $x_{1}[n]$; (**c**,**d**) angle random walk of $x_{2}[n]$.

The coefficient of the angle random walk (*N*) denotes the magnitude of the white noise [30]. According to the 2D description method of noise terms mentioned in Section 4.2, the coefficient *N*(*t*) of the Angle random walk can be acquired. Figure 8 shows the time-varying parameter *N*(*t*) of the two sets’ simulation data. Figure 8a,c shows the results of the classical DAVAR while Figure 8b,d shows the results of the improved fast DAVAR. The coefficient *N*(*t*) of $x_{1}[n]$ fluctuates around a constant while $x_{2}[n]$ increases linearly with time. The results prove that the improved fast DAVAR is correct.

Both the results are obtained by the Matlab program on an Intel(R) Core(TM) i7-3770 CPU with a 3.4 GHz clock. Table 2 shows a computational comparison of the classical DAVAR and the improved fast DAVAR.

In the second column, the number of simulation data points is reported. The third column shows the length of the truncation window and the fourth column shows the step width. The fifth column represents the computational time. The last column indicates the calculation times of the Allan variance in the whole calculation process.

When the length of the time series is short ($6\times 10^{3}$ samples), the improved fast DAVAR saves 78.93% of computing time. When the length of the time series is long ($6\times 10^{5}$ samples), the improved fast DAVAR costs only 27.92727 s while the classical DAVAR costs 960.422362 s. The improved fast DAVAR reduces 97.09% of computing time. The last column reports the reason. With the time series increasing, the classical DAVAR needs to calculate the Allan variance more and more times while the improved fast DAVAR just needs to compute it one time (the initial value of the DAVAR). Thus, the improved fast DAVAR could shorten the calculation time. When the time series is long, the improved fast DAVAR is meaningful and significant.

**Figure 9:**
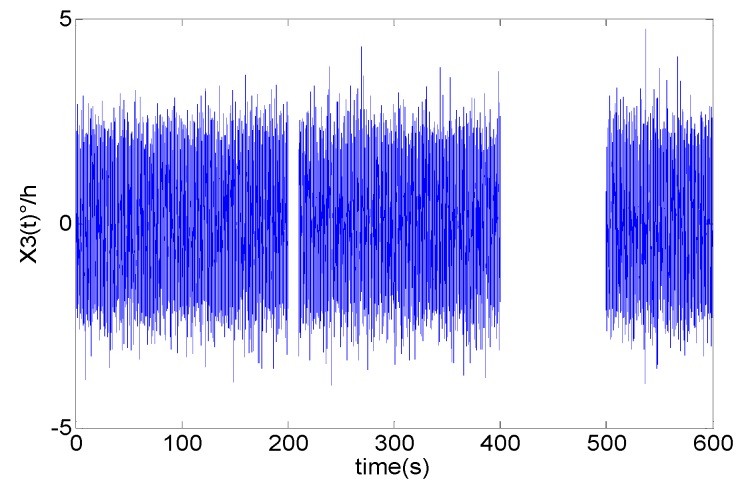
The discontinuous series time.

Now we extend the improved fast DAVAR to discontinuous time series. On the basis of the simulation data $x_{1}[n]$, we created simulation dataset $x_{3}[n]$ using the same noise model as $x_{1}[n]$, but it is longer than $x_{1}[n]$. Two pieces of data in $x_{3}[n]$ are deleted. One piece of data is between 200 s and 220 s and the other piece of data is between 400 s and 500 s. The discontinuous simulation data is represented in Figure 9.

**Figure 10:**
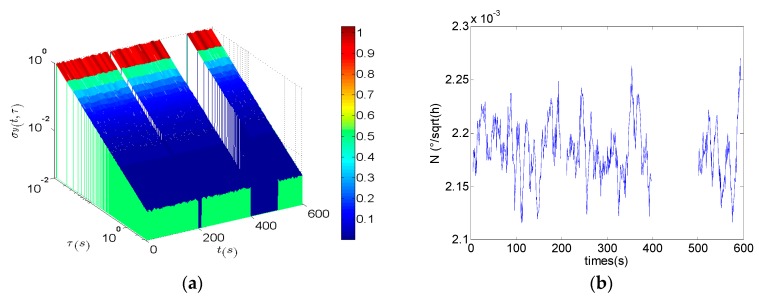
The fast DAVAR of the discontinuous data. (**a**) The improved fast DAVAR of $x_{3}[n]$; (**b**) Angle random walk of $x_{3}[n]$.

Then the classical DAVAR and the improved fast DAVAR are applied to analyzing the simulation data. The classical DAVAR is forced to stop because the program could not identify the NaN. However, the improved fast DAVAR could successfully analyze the data. Figure 10a represents fast DAVAR obtained with a window of $N_{W}=1000$ samples and a step whose width is 30 samples. When *t* < 200 s, the DAVAR is essentially stationary. The slope of the DAVAR surface correctly indicates the presence of white phase noise. In the time interval 200 s < *t* < 220 s, the DAVAR shows a little canyon corresponding to a few areas of missing data. In the region of the large gap 400 s < *t* < 500 s, the surface of the DAVAR exhibits a large canyon corresponding to a greater amount of missing data. Figure 10b shows the coefficient of the angle random walk noise. The coefficient *N*(*t*) of $x_{3}[n]$ is similar to Figure 8a. Moreover, it has the same order of magnitude and the same shape as that of the continuous data besides the two canyons. With this geometrical representation, the fast DAVAR clearly describes the dynamic instability of a gyroscope with missing data.

## 8. Experiments

**Figure 11:**
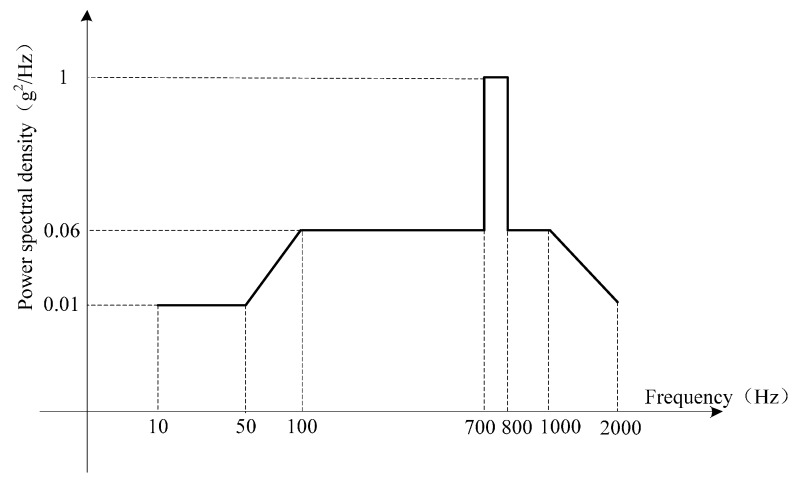
PSD of the random vibration.

Aiming to further verify the proposal algorithm, a vibration experiment was implemented with a ready-made FOGs-based MWD. The accuracy of FOGs in MWD system is 0.03 °/h. The vibration experiment is carried out at the high temperature of 65 °C. The x-axis FOG is mounted along with the vibration direction of the vibration platform. The PSD of the vibration is reported in Figure 11. Its vibration level is high, which simulates the drilling vibration underground. When the vibration frequency is between 700 Hz and 800 Hz, the PSD of the vibration is up to 1 g^2^/Hz. The root-mean-square value of the whole vibration is 13.39 g.

**Figure 12:**
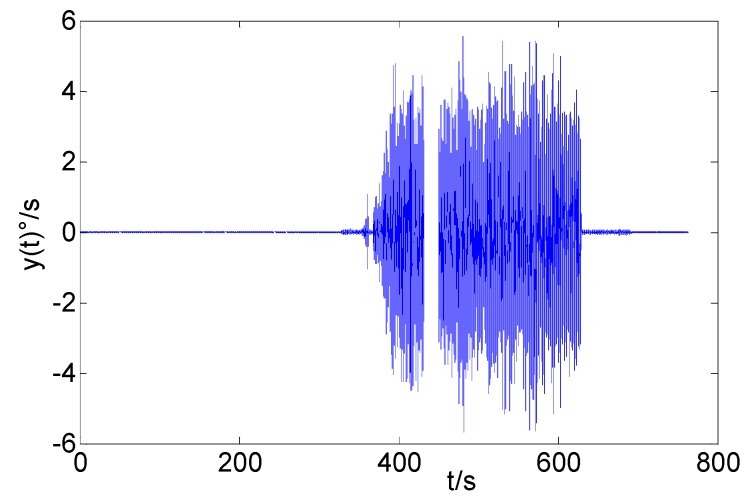
Output signal of x-axis FOG.

The vibration experiment was conducted via the following steps. First, the vibration platform was kept static (0–330 s). Secondly, the vibration platform began to vibrate and kept vibrating for 5 min (330 s–630 s). Finally the vibration platform returned to static state (630 s–930 s). The movement of the MWD system was in accordance with the movement of the vibration platform. We collected the output signal of the x-axis FOG in the MWD system. The sampling interval is 2.5 ms. Hence, the total value of this gyro data is $37.2\times 10^{4}$. The primary signal is shown in Figure 12. It shows that the platform began to vibrate at *t* = 330 s and stopped at *t* = 630 s. In addition, we must pay attention to a piece of lost data when 432 s < *t* < 450 s.

Then the improved fast DAVAR has been applied to analyzing this discontinuous vibration data. Its result is obtained with a truncation window of length $N_{W}=2000$ samples, and a step width of 1000 samples. The Matlab program calculation is carried out on an Intel (R) Core (TM) i7-3770 CPU with a 3.4 GHz clock. The improved fast DAVAR costs only 28.616520 s to deal with this long time series.

**Figure 13:**
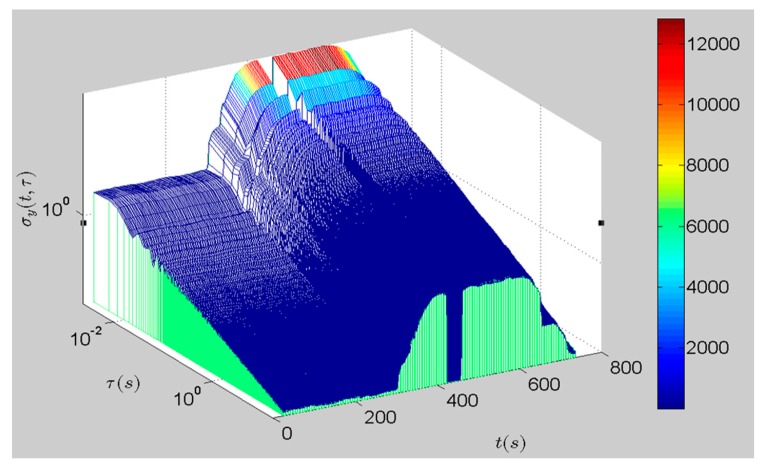
The improved fast DAVAR of the vibration data.

Figure 13 is the improved fast DAVAR result. It can be seen that the DAVAR surface is stationary at the beginning. Then there appears a large crest that starts at *t* = 330 s and stops at *t* = 630 s. In the end it goes back to being stationary. Obviously, the canyon (430 s < *t* < 450 s) is the graphical representation of the missing data in the time series. The changing process of the DAVAR surface is consistent with Figure 12. In conclusion, the improved fast DAVAR could track and reveal the non-stationary characteristics in a clear way.

**Figure 14:**
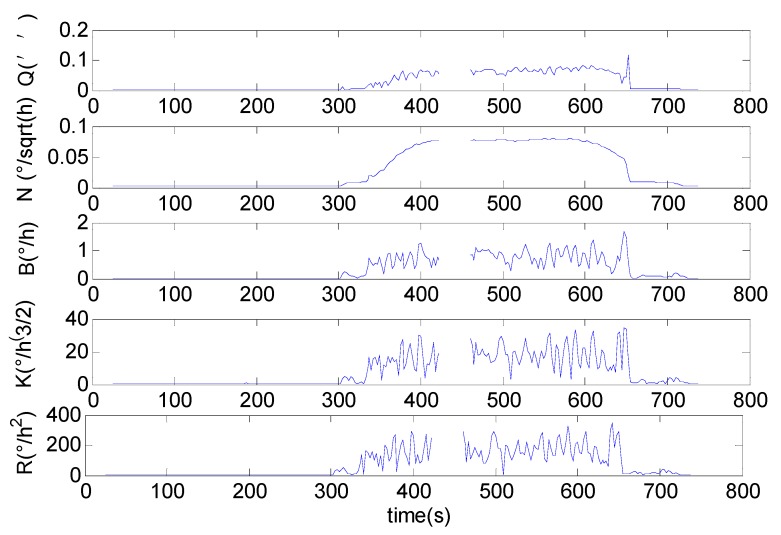
The coefficients of each noise term.

Fitting the double logarithmic curve $\sigma^{2}(t,\tau )-\tau$ at any given time *t*, the time-varying coefficients of noise terms can be obtained [27,28]. Figure 14 shows the changing coefficients of each noise terms evaluated by the improved fast DAVAR.

Aside from the canyon at *t* = 432 s, it is easy to distinguish the change in the noise. Before vibration (*t* < 330 s), since the FOG is static, the coefficient of each noise is small without evident change. When the platform starts to vibrate (*t* = 432 s), the noise terms of the FOG change dramatically, especially the Rate Random walk, which is affected by the vibration. Meanwhile, the coefficient of the quantization noise increases when the FOG is vibrating. The data acquisition circuit of FOG has better aseismic performance. After vibration, all confidents of noise terms are back to the previous state. We can make a conclusion that the FOG could endure the drilling vibration. In conclusion, the improved fast algorithm of the DAVAR could successfully analyze the discontinuous gyroscope data. The improved fast DAVAR identifies and reveals the highly dynamic instability in the FOG’s discontinuous time series.

## 9. Conclusions

The working environment of FOGs-based MWD is hostile: the vibration is very strong and the temperature is very high. The gyroscope is heavily influenced by factors such as temperature, vibration, aging, and sudden breakdowns. A slight variation of the gyroscope stability can turn into a measurement error. Hence, it is important to monitor the behavior of gyroscopes through the use of DAVAR.

The DAVAR is a representative of the time-varying stability of the gyroscope. The fast DAVAR is a fast algorithm for the classical DAVAR. However, both the fast DAVAR and the classical DAVAR could not analyze the discontinuous gyroscope data. What is worse, in many applications, the gyroscope often gives discontinuous data, for example in a FOGs-based MWD system.

In this paper, utilizing the recursive characteristic of the fast DAVAR, we make a further advance on the fast algorithm to extend the fast DAVAR to discontinuous gyroscope data. This not only dramatically reduces the computation time, but could also allow us to analyze the discontinuous gyroscope data. Both the simulation results and the experimental results show that the improved fast DAVAR could not only save more than 90% of the computational time, but also deal successfully with discontinuous data.

## Figures and Tables

**Table 1:** Representation of noise terms using Allan variance.

Noise Terms	Noise Coefficient	$S_{\Omega }(v)$ PSD of the Random Process	$\sigma^{2}(\tau )$	Slope of logσ(τ)-logτ
**the quantization noise**	*Q*	$S_{\Omega }(f)=\{\begin{array}{cc} \frac{4Q^{2}}{\tau }\sin^{2}\;(\pi f\tau ) & f\geq \frac{1}{2\tau }\\ {\left(2\pi f\right)}^{2}\tau Q^{2} & f<\frac{1}{2\tau } \end{array}$	$\frac{3Q^{2}}{\tau^{2}}$	−1
**angular random walk**	*N*	$S_{\Omega }(f)=N^{2}$	$\frac{N^{2}}{\tau }$	−1/2
**bias instability**	*B*	$S_{\Omega }(f)=\{\begin{array}{cc} (\frac{B^{2}}{2\pi })\frac{1}{f} & f\leq f_{0}\\ 0 & f>f_{0} \end{array}$	$\frac{2B^{2}}{\pi }\ln2$	0
**rate random walk**	*K*	$S_{\Omega }(f)=(\frac{K^{2}}{2\pi })\frac{1}{f^{2}}$	$\frac{K^{2}\tau }{3}$	1/2
**the rate slope**	*R*	$S_{\Omega }(f)=\frac{R^{2}}{{\left(2\pi f\right)}^{3}}$	$\frac{R^{2}\tau^{2}}{2}$	1

**Table 2:** The computational comparison.

Data	Numbers	$N_{W}$	Step Width	Time (s)		Computation Times of the Allan Variance	
				DAVAR	Fast DAVAR	DAVAR	Fast DAVAR
*x*_1_[*n*]	$6\times 10^{3}$	1000	30	3.656864	0.770367	166	1
*x*_2_[*n*]	$6\times 10^{5}$	2000	300	960.422362	27.92727	1993	1
